# Optimal timing and clinical value of radiotherapy in advanced *ALK*-rearranged non-small cell lung cancer with or without baseline brain metastases: implications from pattern of failure analyses

**DOI:** 10.1186/s13014-019-1240-1

**Published:** 2019-03-13

**Authors:** Jianjiao Ni, Guodong Li, Xi Yang, Li Chu, Jialei Wang, Yida Li, Liqing Zou, Yuan Li, Congying Xie, Zhengfei Zhu

**Affiliations:** 1 Department of Radiation Oncology, Fudan University Shanghai Cancer Center, 270 Dong An Road, Shanghai, 200032 China; 2 Department of Interventional Radiology, Fudan University Shanghai Cancer Center, Shanghai, China; 3 Department of Medical Oncology, Fudan University Shanghai Cancer Center, Shanghai, China; 4 Department of Pathology, Fudan University Shanghai Cancer Center, Shanghai, China; 5 Department of Oncology, Shanghai Medical College, Fudan University, Shanghai, 200032 China; 6 Radiotherapy and Chemotherapy Department, the 1st Affiliated Hospital of Wenzhou Medical University, Wenzhou, China

**Keywords:** Non-small cell lung cancer, ALK, Radiotherapy, Pattern of failure, Brain metastases, Survival

## Abstract

**Background:**

Despite development of several next-generation tyrosine kinase inhibitors (TKIs), crizotinib remains one of the first-line treatment options for advanced *ALK*-positive NSCLC and is widely used in situations where next-generation TKIs aren’t yet approved or economically inaccessible. However, the pattern of failure and clinical value of radiotherapy in metastatic crizotinib-treated *ALK*-mutant lung cancer, with or without baseline brain metastases (BBM), are largely unknown.

**Methods:**

Consecutive crizotinib-treated NSCLC patients with adequate imaging and measurable disease were retrospectively enrolled. Disease progression in original sites (primary/metastatic), new sites, or both, are classified as original failure (OF), distant failure (DF) and ODF, respectively. Progression free survival, from crizotinib initiation to the first disease progression, and from that to the second disease progression, were calculated as PFS1 and PFS2.

**Results:**

Ninety-three patients were identified. With a median follow up of 22.0 (range, 2.0–72.0) months, 52 patients had crizotinib-treatment failure. The frequencies of OF, ODF, and DF, were 50.0, 26.9, and 23.1%, respectively. Histology, primary tumor size and presence of BBM, were independently associated with OF, using competing risks analyses. The brain was the most common site of initial disease progression. Patients with BBM had a significant higher possibility developing multiple-progressive lesions in the brain (*p* = 0.002). Importantly, four of the ten patients who had baseline oligo-metastatic cranial disease but didn’t receive upfront brain radiation, developed multiple-progressive disease in the brain. Brain radiation before crizotinib could alter the disease failure patterns and improve PFS1 among patients with BBM (*p* = 0.006). Extracranial radiation was efficient in controlling symptoms but it was not associated with PFS1 (*p* = 0.223), and the majority of patients were eligible for salvage radiotherapy upon disease progression to crizotinib. By the time of data cut-off, 28 patients had second disease progression, with a median PFS2 of 7.0 (95% CI 5.4–8.6) months and salvage radiotherapy significantly prolonged PFS2 (*p* = 0.003). Additionally, patients receiving any radiotherapy during their treatment course had a significant longer overall survival (*p* = 0.048).

**Conclusions:**

Among patients with baseline oligo-metastatic brain lesions which are suitable for stereotactic radiosurgery, upfront brain radiotherapy provides considerable clinical benefits. While, extracranial radiation may be deferred in asymptomatic patients with multiple-metastatic lesions.

**Electronic supplementary material:**

The online version of this article (10.1186/s13014-019-1240-1) contains supplementary material, which is available to authorized users.

## Introduction

Lung cancer is the leading cause of cancer-related deaths across the world [1]. Anaplastic lymphoma kinase (ALK) rearrangements lead to an in-frame fusion protein with oncogenic activity and are detected in approximately 5% of non-small cell lung cancer (NSCLC) cases [2, 3]. Crizotinib was the first tyrosine kinase inhibitor (TKI) targeting ALK and demonstrated remarkable efficacy against *ALK*-positive lung cancer [4, 5]. Generally, crizotinib treatment is associated with a median progression-free survival (PFS) of 7–11 months and an objective response rate of approximately 60%, which is significantly superior to chemotherapy in both treatment-naïve and pretreated patients [5, 6]. However, disease progression ultimately occurs, and the molecular mechanisms underlying crizotinib resistance are currently being investigated [7, 8]. However, elaborate analyses of patterns of crizotinib-treatment failure in these patients to examine the feasibility of radiotherapy has not been performed.

Brain metastases occur in up to 60% of *ALK*-positive NSCLC and lead to considerable morbidity and mortality [9, 10]. Compared with chemotherapy, crizotinib demonstrated a higher intracranial response rate and a longer intracranial PFS [5, 6]. However, the brain is the most common site of disease progression during crizotinib treatment, and up to 70% of patients with baseline brain metastases (BBM) develop treatment failure in the central nervous system [9, 10]. Among patients with advanced *ALK*-positive NSCLC, tumor characteristics in the central nervous system, patterns of disease evolution in the brain, and eligibilities for stereotactic radiotherapy, in the baseline or after certain treatment, have not been fully elucidated.

The clinical value of radiotherapy for patients with metastatic *ALK*-positive NSCLC remains controversial. Recently, synergistic effects between radiation and TKIs are demonstrated [11, 12] and under certain circumstance, radiotherapy may improve survival among advanced oncogene-driven NSCLC [13, 14]. In addition, despite development of several next-generation TKIs targeting ALK rearrangement with higher potency and greater blood-brain-barrier penetrating capacity, crizotinib remains one of the first-line treatment options for advanced ALK-positive NSCLC [15] and is widely used in situations where next-generation TKIs are not yet approved or economically inaccessible. Therefore, to investigate the real-world outcomes of radiotherapy in crizotinib-treated advanced *ALK*-positive NSCLC, are still having great significance.

## Materials and methods

### Patients

We reviewed the medical records of crizotinib-treated metastatic *ALK*-rearranged NSCLC patients treated between January 2014 and March 2018 at Fudan University Shanghai Cancer Center. The inclusion criteria were patients with pathologically-confirmed NSCLC, and ALK rearrangements verified by the break-apart fluorescent in situ hybridization assay. Complete serial imaging was collected and reviewed by two independent experienced radiologists. Finally, patients with adequate radiological data and measurable disease were enrolled.

Clinical data, including time of cancer diagnosis, age at diagnosis, sex, tumor histology, primary tumor size, tumor stage, Eastern Cooperative Oncology Group (ECOG) score, numbers and sites of metastatic disease, characteristics and timing of treatment modalities, status and time of disease progression, status and time of death, and time of most recent follow-up were collected from electronic medical records. Data were cut-off by May 31, 2018. This study was approved by the institutional review board of Fudan University Shanghai Cancer Center.

### Pattern of failure analysis

Baseline and follow-up imaging, which was typically contrast-enhanced computed tomography (CT) of the chest, abdomen, and pelvis, with or without positron emission tomography, were obtained for all patients. Follow-up scans were typically performed every 6–8 weeks. Baseline brain scans were performed with either CT or magnetic resonance imaging (MRI). For patients without BBM, follow-up brain scans were not mandatory and were performed at the discretion of treatment teams. Treatment responses were assessed using Response Evaluation Criteria in Solid Tumors (RECIST) 1.1 guidelines.

The development of progressive disease within primary and/or metastatic lesions that were present before crizotinib was defined as original failure (OF). The occurrence of a new metastatic lesion that was not identified before crizotinib was defined as distant failure (DF). The simultaneous development of OF and DF was defined as ODF.

### Statistical analysis

To identify potential predictors of OF, we performed univariate and multivariate analyses of associations between common clinical-pathological parameters with time to progression, using competing risk methodology and Stata version 13.1 software (StataCorp, College Station, TX, USA), with DF and ODF both defined as competing events.

PFS and overall survival (OS) were calculated using RECIST1.1 criteria. PFS1 was calculated from the initiation of crizotinib to the first disease progression or any-cause of death. PFS2 was calculated from the time of first disease progression to the second disease progression or any-cause of death. OS was calculated from the time of diagnosis to any-cause of death. The Kaplan-Meier method was used to estimate survival, and differences among groups were investigated by the log-rank test. Cox proportional hazards models were employed to calculate hazard ratios (HRs) and 95% confidence intervals (CIs), between covariates and survival. χ2 or Fisher’s exact tests were used to compare qualitative data. These analyses were performed using SPSS version 22.0 software (IBM, Armonk, NY, US). All statistical tests were two-sided, and *p* < 0.05 was defined as statistically significant.

## Results

### Patient characteristics

Between January 2014 and March 2018, 138 patients with metastatic *ALK*-rearranged NSCLC received crizotinib and had regular follow-ups at Fudan University Shanghai Cancer Center. Complete serial images of these patients were examined, and 93 patients with adequate radiological data and measurable disease were identified. Detailed clinical features of the 93 patients are shown in Table 1.

**Table 1:** Disease characteristics among patients who received radiotherapy during their treatment course (RT) and who didn’t (Non-RT), as well as in the whole population

	ALL patients n (%)	Non-RT n (%)	RT n (%)	*p*
Age (years)				0.958
Median	50	49	50	
Range	21–75	28–75	21–68	
Sex				0.079
Male	55 (59.1)	26 (51.0)	29 (69.0)	
Female	38 (40.9)	25 (49.0)	13 (31.0)	
Histology				0.452
Adenocarcinoma	90 (96.8)	50 (98.0)	40 (95.2)	
Non-adenocarcinoma	3 (3.2)	1 (2.0)	2 (4.8)	
ECOG				0.814
0–1	88 (94.6)	48 (94.1)	40 (95.2)	
2	5 (5.4)	3 (5.9)	2 (4.8)	
Primary tumor Size (cm)				0.390
Median	3.5	3.5	3.0	
Range	0.0–8.0	0.0–8.0	0.0–7.9	
T stage				0.841
T1–2	52 (55.9)	29 (56.9)	23 (54.8)	
T3–4	41 (44.1)	22 (43.1)	19 (45.2)	
N stage				0.513
N0	9 (9.7)	4 (7.8)	5 (11.9)	
N1–3	84 (90.3)	47 (92.2)	37 (88.1)	
Number of metastatic organs				0.055
1–2	66 (71.0)	32 (62.7)	34 (81.0)	
≥ 3	27 (29.0)	19 (37.3)	8 (19.0)	
Number of metastatic lesions				0.053
1–5	28 (30.1)	11 (21.6)	17 (40.5)	
≥ 6	65 (69.9)	40 (78.4)	25 (59.5)	
Presence of lung metastases				0.069
Yes	24 (25.8)	17 (33.3)	7 (16.7)	
No	69 (74.2)	34 (67.7)	35 (83.3)	
Presence of liver metastases				0.119
Yes	15 (16.1)	11 (21.6)	4 (9.5)	
No	78 (83.9)	40 (78.4)	38 (90.5)	
Presence of brain metastases				0.010
Yes	35 (37.6)	13 (25.5)	22 (52.4)	
No	58 (62.4)	38 (74.5)	20 (47.6)	
Presence of adrenal metastases				0.086
Yes	13 (14.0)	10 (19.6)	3 (7.1)	
No	80 (86.0)	41 (80.4)	39 (92.9)	
Presence of Bone metastases				0.698
Yes	33 (35.5)	19 (37.3)	14 (33.3)	
No	60 (64.5)	32 (62.7)	28 (66.7)	

*ECOG* Eastern Cooperative Oncology Group

Before crizotinib, brain and bone were the most common metastatic sites followed by lung, liver, adrenal gland, and other sites. The clinical features of the 35 patients with BBM were generally consistent with the whole population. Brain metastases with no more than three lesions and a maximum size of lesions < 3 cm is considered oligo-metastatic disease, the others are considered multiple-metastatic diseases. Among the 35 patients with BBM, 15 (42.9%) had oligo-metastatic disease, while the remaining 20 patients had multiple-metastatic disease.

### Crizotinib treatment and PFS1

All patients received crizotinib (Xalkori, Pfizer, La Jolla, CA, USA) starting at 250 mg twice a day orally. Fifty-seven patients received crizotinib as first-line therapy, while the remaining 36 had one or two lines of prior chemotherapy, with or without prior radiotherapy. In fact, 25 patients received radiation before crizotinib. Brain radiation was administered to 11 patients, thoracic radiation to 10, bone radiation to five, and adrenal gland radiation to on patient. Regarding brain radiation, stereotactic radiosurgery (SRS) was performed in all of the cranial lesions in the four patients with oligo-metastatic disease and to the largest three cranial lesions in a patient with multiple-metastatic disease. Additionally, whole brain radiotherapy (WBRT) was performed in five patients with multiple-metastatic disease and one patient with oligo-metastatic disease. WBRT was administered at 30Gy/10 fractions, while brain SRS was performed typically at a dose of 18-20Gy per lesion. Of note, 4 out of the six patients receiving WBRT were symptomatic and brain radiation led to a significant symptom alleviation in 3 patients. The detailed information of extracranial radiation was summarized in Additional file 1: Table S1.

After crizotinib treatment, a complete response occurred in one patient, partial responses in 66 patients, stable disease in 22 patients, and progressive disease in four patients, yielding an objective response rate of 72.0% and a disease control rate of 95.7%. With a median follow-up of 22.0 months (range: 2.0–72.0 months), disease progression was documented in 52 patients, and the median PFS1 was 11.5 months (95%CI: 9.8–13.2 months). Univariate and multivariate analyses between associations of common clinical-pathological parameters with PFS1, were performed. Histology of adenocarcinoma (*p* = 0.040), having less than three metastatic organs (*p* = 0.005), and achieving objective response to crizotinib (*p* = 0.036), were independently associated with longer PFS1, in multivariate analysis. In contrast, neither prior radiation (*p* = 0.223) nor the presence of BBM (*p* = 0.089), were significantly associated with PFS1.

**Fig. 1:**
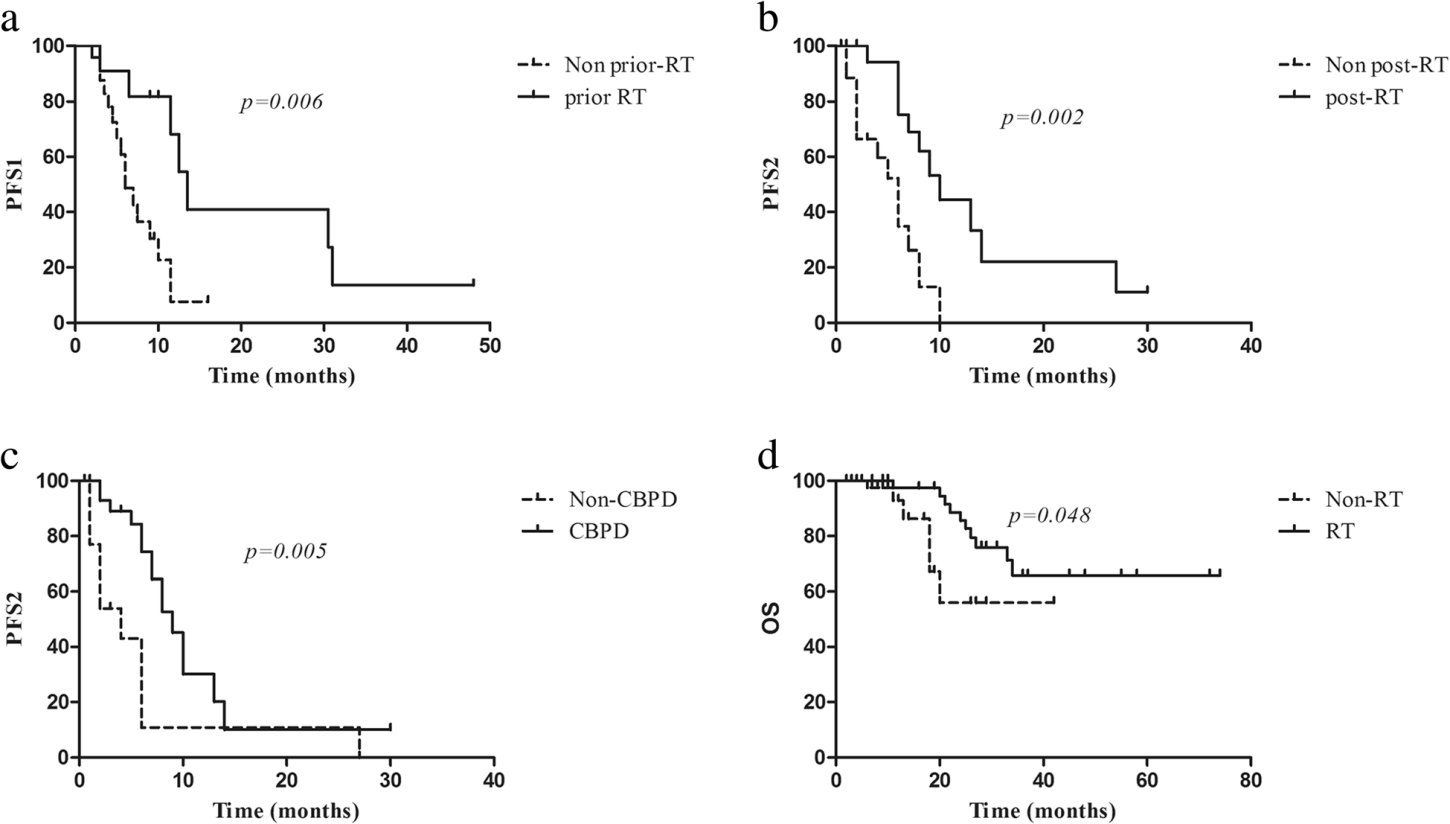
Kaplan-Meier survival curves. **a** among patients with baseline brain metastases, comparing patients who received brain radiotherapy prior to crizotinib (prior-RT) versus those who did not (Non prior-RT), **b** among patients with crizotinib treatment failure, comparing patients who received salvage radiation post initial disease progression (post-RT) versus those who did not (Non post-RT), **c** among patients with crizotinib treatment failure, comparing patients who continued crizotinib administration beyond progressive disease (CBPD) versus those who did not (Non-CBPD), **d** comparing patients who received radiotherapy during their treatment course (RT) versus those who did not (Non-RT), in the whole population

The median PFS1 for patients with BBM was 10.0 months (95% CI: 6.9–13.1 months). As shown in Fig. 1a, compared with those who had BBM but did not receive prior brain radiotherapy, the 11 patients who had BBM and received prior brain radiotherapy had significant longer PFS1 (median PFS1 6.0 vs 13.5 months, respectively; *p* = 0.006).

### Pattern of failure analysis and impact of prior radiotherapy

**Fig. 2:**
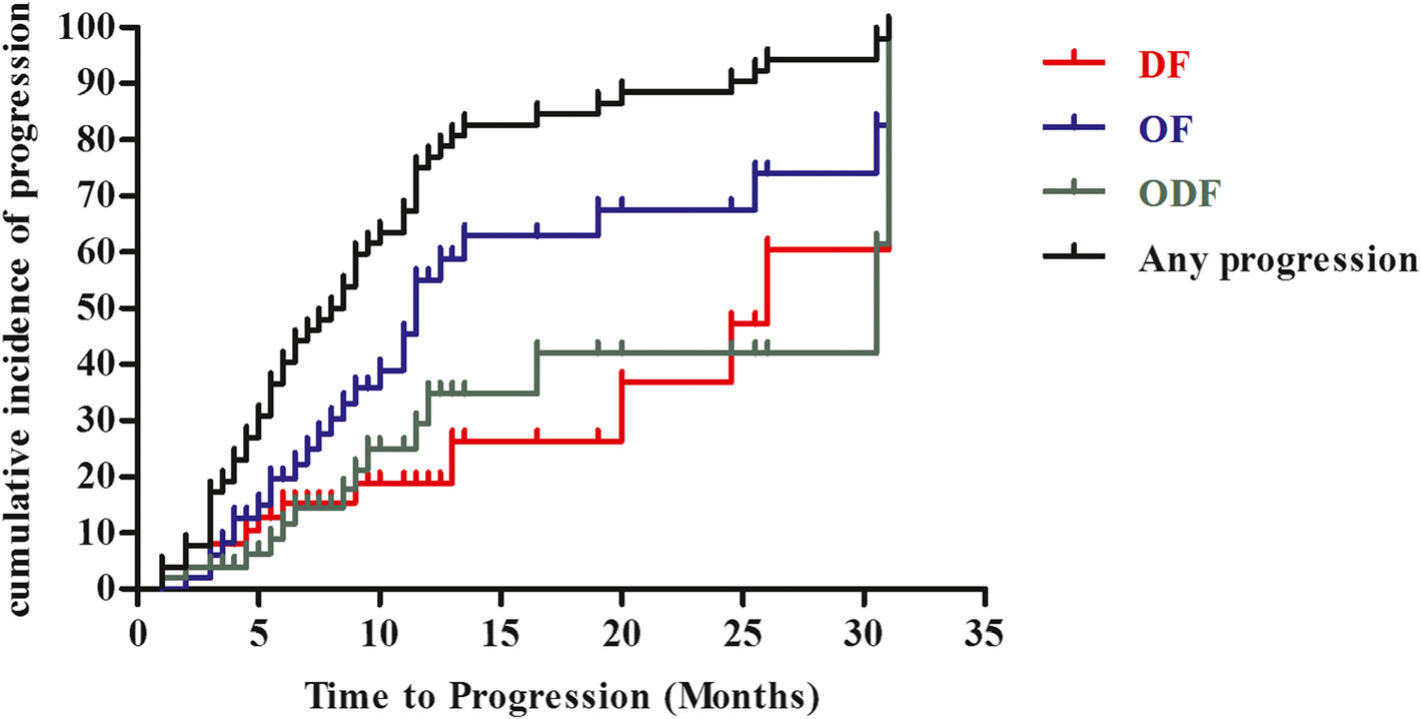
Pattern of failure analysis. The cumulative incidence of any progression and each of the three patterns of failure, original site failure (OF), distant site failure (DF), and simultaneous OF/DF (ODF) as a function of follow-up time

The initial sites of disease progression are shown in Table 2. Half of the patients developed OF, 14 developed ODF, and the remaining 12 developed DF. The cumulative actuarial rates of any progression at 12 and 24 months were 76.9 and 90.4%, respectively (Fig. 2).

**Table 2:** Sites of initial failure

	All patients (*n* = 52)	Patients with OF (*n* = 26)	Patients with DF (*n* = 12)	Patient with ODF (*n* = 14)
	n (%)	n (%)	n (%)	n (%)
Initial PD				
Primary only	9 (17.3)	7 (26.9)	0 (0.0)	0 (0.0)
Metastasis only	33 (63.5)	16 (61.5)	12 (100.0)	7 (50.0)
Primary and metastasis	10 (19.2)	3 (11.5)	0 (0.0)	7 (50.0)
Site of initial failure				
Lung	17 (32.7)	7 (26.9)	2 (16.7)	8 (57.1)
Liver	3 (5.8)	2 (7.7)	0	1 (7.1)
Brain	31 (59.6)	15 (57.7)	6 (50.0)	10 (71.4)
Bone	6 (11.5)	1 (3.8)	3 (25.0)	2 (14.3)
Adrenal grand	1 (1.9)	0 (0.0)	0	1 (7.1)
Regional LN	6 (11.5)	3 (11.5)	0	3 (21.4)
Distant LN	4 (7.7)	2 (7.7)	0	2 (14.3)
others	3 (5.8)	1 (3.8)	1 (8.3)	1 (7.1)

*OF* original site failure, *DF* distant site failure, *ODF* simultaneous occurrence of OF and DF, *n* number, *PD* disease progression, *LN* lymph node

Brain was the most common site of initial disease progression, followed by the lung and bone. Thirty (57.7%) patients developed initial progressive disease in the brain, with or without extra-cranial progressive disease. Seventeen (48.6%) of the 35 patients with BBM, as well as 13 (22.4%) of the 58 without BBM, developed progressive disease in the brain. Hence, patients with BBM had a higher risk of developing progressive disease in the brain (48.6% vs 22.4%; *p* = 0.012). Furthermore, similar to baseline brain metastases, disease progression in the brain was classified into two categories: cranial progressive disease with no more than three progressive lesions with a maximum size of < 3 cm was classified as oligo-progressive disease. The others were classified as multiple-progressive diseases. Among the 17 patients with BBM who developed progressive disease in the brain, 15 (88.2%) developed multiple-progressive disease, while among the 13 patients without BBM who developed progressive disease in the brain, only four (30.8%) developed multiple-progressive disease. Thus, patients with BBM had a higher risk of developing multiple-progressive disease in the brain (88.2% vs 30.8%; *p* = 0.002).

**Fig. 3:**
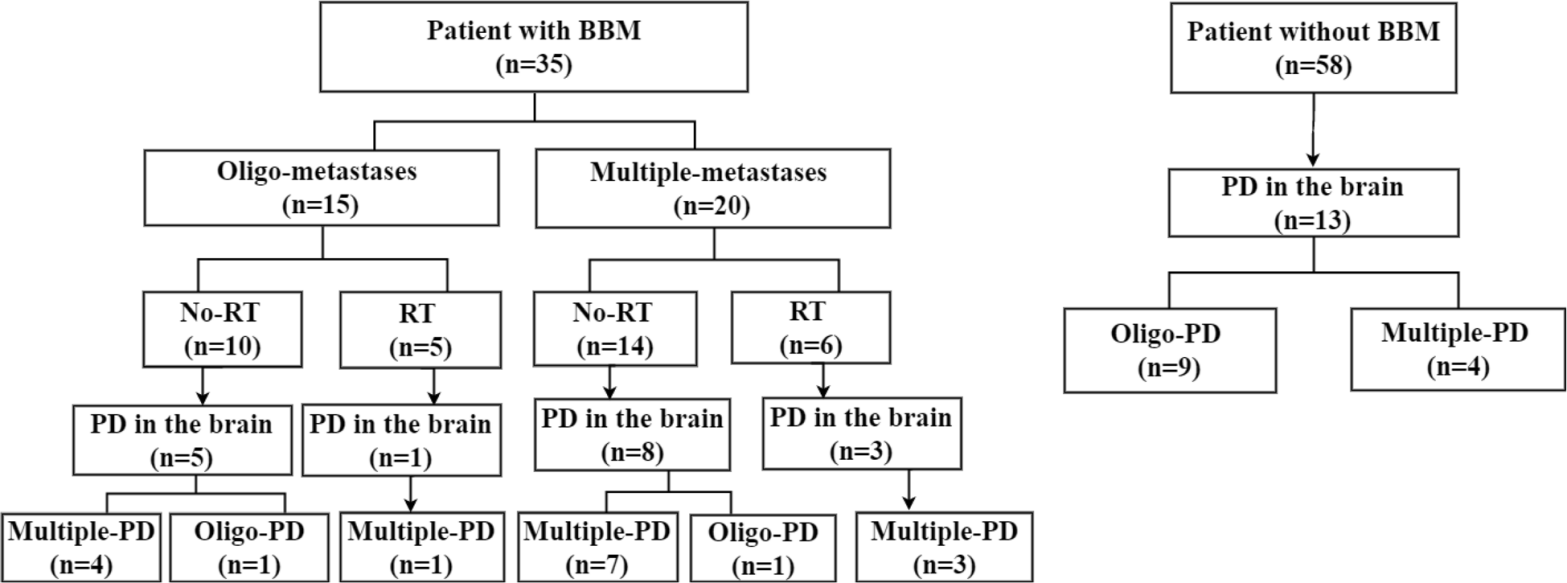
Disease evolution in the brain under crizotinib treatment. BBM, baseline brain metastases; PD, progressive disease; RT, radiotherapy; oligo-PD, oligo-progressive disease; multiple-PD, multiple-progressive disease

We also found that the patterns of cranial disease progression were altered by prior radiotherapy. In fact, among the 11 patients with BBM who were treated with radiotherapy prior to crizotinib, only four developed progressive disease in the brain. In contrast, among the 24 patients with BBM who did not receive prior radiotherapy, 13 developed progressive disease in the brain. More importantly, four of the ten (40.0%) patients who had baseline oligo-metastatic cranial disease but did not receive prior brain radiation, developed multiple-progressive disease in the brain. In contrast, none of the four patients who had baseline oligo-metastatic cranial disease and received prior brain SRS, developed multiple-progressive disease in the brain. The evolutions of cranial disease of the whole population were summarized in Fig. 3.

The median time to maximum response, median PFS1, and response rate were not significantly different among patients with OF, ODF, or DF. Using the actuarial competing risks methodology in univariate analysis, histology, primary tumor size, number of metastatic organs, number of metastatic lesions and presence of BBM were found to be associated with OF. Meanwhile, non-adenocarcinoma histology, primary tumor size > 3.5 cm, and presence of BBM remained independent predictors of OF in multivariate analysis (Table 3).

**Table 3:** Predictors of Original Site Failure

	Univariate Analysis		Multivariate Analysis	
	SHR	*p*	SHR	*p*
Age (≤50y vs >50y)	0.55 (0.26–1.16)	0.116		
Sex (female vs male)	0.61 (0.29–1.26)	0.183		
Histology (non-adenocarcinoma vs adenocarcinoma)	0.19 (0.08–0.43)	< 0.001	0.19 (0.05–0.78)	0.021
ECOG (0–1 vs 2)	0.45 (0.12–1.75)	0.250		
Primary tumor size (< 3.5 vs ≥3.5)	2.74 (1.28–5.87)	0.010	2.45 (1.12–5.35)	0.024
T stage (T1–2 vs T3–4)	0.88 (0.42–1.85)	0.749		
N stage (N0 vs N1–3)	3.13 (0.68–14.5)	0.144		
Number of metastatic organs (1–2 vs ≥3)	2.44 (1.14–5.24)	0.022	1.77 (0.78–4.04)	0.175
Number of metastatic lesions (1–5 vs ≥6)	4.16 (1.29–13.5)	0.017	3.12 (0.77–12.7)	0.111
Presence of lung metastases (No vs Yes)	0.84 (0.33–2.10)	0.703		
Presence of liver metastases (No vs Yes)	1.57 (0.06–4.39)	0.386		
Presence of brain metastases (No vs Yes)	3.50 (1.62–7.54)	0.001	2.31 (1.07–4.97)	0.033
Presence of adrenal metastases (No vs Yes)	1.59 (0.51–4.95)	0.423		
Presence of bone metastases (No vs Yes)	1.13 (0.51–2.37)	0.737		
Prior lines of therapy (≥1 vs 0)	1.02 (0.48–2.10)	0.960		
Prior local therapy (No vs Yes)	1.13 (0.55–2.32)	0.731		
Objective response (No or Yes)	0.84 (0.36–1.95)	0.691		

*ECOG* Eastern Cooperative Oncology Group

### Salvage radiotherapy, PFS2 and OS

After RECIST-defined treatment failure to crizotinib, the feasibility of salvage radiotherapy for progressive lesions in each patient was rigorously examined. Generally, patients with multiple-progressive disease in the brain and had not received prior WBRT were considered suitable for WBRT. Patients with oligo-progressive disease in the brain were considered eligible for brain SRS. Patients with extra-cranial progressive disease who met the criteria for stereotactic body radiation therapy (SBRT), as adapted from Al-Hallaq et al. [16], were considered feasible for SBRT. As a result, 35 (67.3%) of the 52 patients with progressive disease were identified as eligible for certain types of subsequent radiotherapy: 19 patients with multiple-progressive disease in the brain were eligible for WBRT, 10 with oligo-progressive disease in the brain were suitable for brain SRS, and 6 with limited progressive disease in extra-cranial sites such as bone and liver were candidates for SBRT.

In fact, 19 patients received salvage radiation after RECIST-defined treatment failure to crizotinib. WBRT was administered in eight patients, brain SRS in five, extra-cranial SBRT in six. By the time of data cut-off, 28 patients had second disease progression, with a median PFS2 of 7.0 months (95% CI: 5.4–8.6 months). As shown in Fig. 1b, compared with patients who did not receive salvage radiation after crizotinib treatment failure, patients who received salvage radiation after RECIST-defined disease progression to crizotinib had significantly longer PFS2 (10.0 vs 6.0 months). Additionally, after RECIST-defined treatment failure to crizotinib, 35 patients continued crizotinib beyond progressive disease (CBPD), with or without concurrent LTs. The other 17 patients discontinued crizotinib, with nine receiving best supportive care, five changing to second-generation ALK inhibitors, and three changing to chemotherapy. Furthermore, As shown in Fig. 1c, CBPD significantly improved PFS2 (9.0 vs 4.0 months).

Finally, by the time of data cut-off, 16 patients had died and 42 had received radiation during their treatment courses. WBRT was performed in 18 patients, brain SRS in 10, thoracic radiation in 14, bone radiation in 8, liver radiation in 2, adrenal gland radiation in 1, and distant lymph node radiation in 1. As shown in Fig. 1d, compared with those who did not receive any radiation during their treatment course (non-RT group), the 42 patients who received certain radiation (RT group), had significantly longer overall survival (*p* = 0.048). Of note, the baseline clinical-pathological parameters between these two groups were generally balanced, except that there were higher percentages of patients with BBM in the RT group (Table 1).

## Discussion

Despite development of several next generation TKIs, crizotinib remain one of the first-line treatment options for advanced *ALK*-positive NSCLC and is widely used across the world. Hence, examining the feasibility and optimal timing of incorporating radiotherapy into crizotinib treatment, in order to improve patients’ quality of life as well as progression-free survival, is still of great significance. To the best of our knowledge, this is the first comprehensive analysis of patterns of crizotinib-treatment failure and the real-world use of radiotherapy in patients with metastatic *ALK*-positive NSCLC. Initial disease progression to crizotinib predominantly occurred in originally-existing lesions, which is consistent with previous studies done in unselected lung cancer populations and in EGFR-mutant patients [17, 18]. These findings provide a rationale for local therapies, especially radiations, directed at existing disease sites.

In this study, brain radiotherapy prior to crizotinib in patients with BBM improved PFS1 and decreased the risk of developing progressive disease in the brain. In a retrospective analysis of patients with asymptomatic BBM using combined data from PROFILE 1005 and PROFILE 1007, brain radiotherapy prior to crizotinib was shown to prolong the intracranial time to progression from 7.0 to 13.2 months, but not overall PFS (5.9 months vs 6.0 months) [10]. In our study, patients with both asymptomatic and symptomatic BBM were included, and more than half of the patients harbored multiple-metastatic disease; thus representing a cohort of patients with higher cranial tumor burden, which is more comparable to the unselected patient populations seen in routine clinical practice [9, 19]. An underlying reason for the clinical benefits of brain radiotherapy prior to crizotinib may be that uncontrolled brain metastases can serve as seeds for new brain metastases, and brain radiotherapy effectively controls cranial metastases. In fact, patients with BBM were found to have a higher risk of developing initial disease progression in the brain, as well as developing multiple-progressive lesions in the brain. Furthermore, 40% of the patients with baseline oligo-metastatic disease who were eligible for brain SRS but did not receive prior brain radiotherapy ultimately developed multiple-progressive disease in the brain. Therefore, deferring brain radiotherapy in patients with baseline oligo-metastatic disease may result in losing the opportunity for brain SRS. Among patients with *ALK*-rearranged NSCLC and brain metastases, initial treatment with SRS or WBRT was shown to induce a similar OS in a multi-institutional study [9]. However, compared with WBRT, brain SRS has been repeatedly demonstrated to show superior efficacies in preserving cognition and improving quality of life [20–22].

Nevertheless, the clinical values of extracranial radiation prior to crizotinib remain controversial. In our study, extracranial radiotherapies were administered before crizotinib in 15 patients, including thoracic radiation, bone radiation, and adrenal gland radiation. They were efficient in mitigating patients’ symptoms, such metastatic bone pain, obstructive pneumonitis and superior vena cava syndrome, which not only improved patients’ quality of life but may also lengthen their ability to receive further therapy. However, extracranial radiation didn’t prolong PFS1 in our study, which is consistent with previous studies demonstrating that palliative radiotherapy can provide safe, effective, and durable symptom control, but generally could not prolong survival, especially in patients with multiple metastases and node-positive disease [23, 24]. In our study, OF was found to be the predominant pattern of crizotinib-treatment failures, but most OF occurred in original metastatic lesions with a diverse distribution of involved organs, making it difficult to select the appropriate organ to target before crizotinib. Moreover, salvage radiotherapies were feasible and beneficial in a considerable percentages of patients after RECIST-defined disease progression. In our study, approximately 66% of patients who progressed on crizotinib were eligible for salvage radiation and patients who indeed received salvage radiotherapies had a significantly longer PFS2. This is in agreement with previous studies that investigated the potential roles of SBRT for patients with limited cranial- and extracranial-progressive disease [13, 25].

There were some limitations to this retrospective study. Next-generation ALK inhibitors are not approved in China by the time of data cut-off of this study and only five patients have received second-generation ALK inhibitors in clinical trials. Hence, the conclusions derived from this study should be interpreted with caution. However, there are preliminary data supporting the administration of brain radiotherapy prior to next-generation TKIs. More than half of the patients with BBM who received first-line ceritinib developed initial progression in the brain [26]. In the ALEX study, patients with BBM receiving first-line alectinib had a 12-month cumulative incidence rate of central nervous system progression, of 8.6 and 20.5%, among those who had received prior brain radiotherapy and those who had not receive prior brain radiotherapy, respectively [27]. In addition, among patients with BBM who received alectinib [28] or brigatinib [29] after failing crizotinib, the cumulative rates of cranial disease progression were still higher than that of extra-cranial disease progression, commonly exceeding 40% at 24 months. On the other hand, accumulating evidence suggest that continuing crizotinib and subsequent administration of proper local therapy, mostly radiation, seem to be a cost-effective treatment strategy among patients with initial crizotinib-treatment failure [30, 31]. Patients who received salvage radiotherapy after crizotinib failure had a median PFS2 of 10 months in our study, which is comparable to the median PFS reported among patients who are subsequently treated with ceritinib [32] or alectinib [33] or brigatinib [34]. By incorporating proper radiotherapies, the duration of crizotinib treatment could be prolonged and next-generation TKIs may be used after the second disease progression, which may translate into overall survival benefit. However, this hypothesis need to be tested in randomized clinical trials.

## Conclusion

Taken together, upfront brain radiotherapy may have considerable clinical advantages, for patients with advanced *ALK*-rearranged NSCLC and baseline oligo-metastatic cranial lesions which are suitable for brain SRS. On the other hand, among asymptomatic patients with multiple-metastatic lesions which are not suitable for SBRT, deferring extracranial radiation until after initial crizotinib-treatment failure and adopting regular surveillance may be a better treatment strategy.

## Additional file

Additional file 1: **Table S1.** Extracranial radiation prior to crizotinib. (DOCX 24 kb)

## Abbreviations

ALK: Anaplastic lymphoma kinase
BBM: Baseline brain metastases
CBPD: Continued crizotinib beyond progressive disease
CI: Confidence intervals
CT: Computed tomography
DF: Distant failure
ECOG: Eastern Cooperative Oncology Group
HR: Hazard ratios
LT: Local therapy
MRI: Magnetic resonance imaging
NSCLC: Non-small cell lung cancer
OF: Original failure
OS: Overall survival
PFS: Progression-free survival
RECIST: Response Evaluation Criteria in Solid Tumors
SBRT: Stereotactic body radiation therapy
SRS: Stereotactic radiosurgery
TKI: Tyrosine kinase inhibitor
WBRT: Whole brain radiotherapy
