# Supplementary material for: Optimal timing and clinical value of radiotherapy in advanced ALK-rearranged non-small cell lung cancer with or without baseline brain metastases: implications from pattern of failure analyses
Source: Radiat Oncol. 2019 Mar 13;14:44. doi: 10.1186/s13014-019-1240-1 (PMC6417092; doi:10.1186/s13014-019-1240-1)
Supplement: Supplementary file 1 — Table S1. Extracranial radiation prior to crizotinib. (DOCX 24 kb) [file 13014_2019_1240_MOESM1_ESM.docx]

**Additional file 1: Table S1**. Extracranial radiation prior to crizotinib

|  | Sex/Age | RT Site | Symptom | Technique | Intention^#^ | Efficacy | Severe Toxicity |
| --- | --- | --- | --- | --- | --- | --- | --- |
| Case1 | M/41 | Thorax | Obstructive pneumonitis | IMRT | Palliative | Symptom  relief | None |
| Case2 | M/41 | Bone | Pain | SBRT | Palliative | Pain relief | None |
| Case3 | F/49 | Bone | Pain | SBRT | Palliative | Pain relief | None |
| Case4 | M/49 | Thorax | Not specific | IMRT | Ablative | Tumor reduction | None |
| Case5 | M/45 | Thorax | Not specific | SBRT | Ablative | Tumor reduction | None |
| Case6 | M/57 | Bone | Pain | SBRT | Palliative | Pain relief | None |
| Case7 | M/53 | Thorax | Not specific | IMRT | Ablative | Tumor reduction | None |
| Case8 | M/39 | Thorax | Not specific | IMRT | Ablative | Tumor reduction | Febrile  neutropenia |
| Case9 | F/38 | Adrenal  gland, Thorax | Not specific | SBRT | Ablative | Tumor reduction | Skin injury |
| Case10 | M/53 | Thorax | Esophageal obstruction | IMRT | Palliative | Symptom  relief | None |
| Case11 | F/58 | Thorax | SVCS | IMRT | Palliative | Symptom  relief | None |
| Case12 | F/53 | Bone | Pain | IMRT | Palliative | Pain relief | None |
| Case13 | F/48 | Bone | Pain, Pathological fracture | IMRT | Palliative | Pain relief | None |
| Case14^*^ | F/50 | Thorax | Not specific | IMRT | Palliative | Tumor reduction | Radiation pneumonitis |
| Case15 | M/52 | Thorax | Not specific | IMRT | Ablative | Stable disease | None |

RT: radiotherapy, IMRT: intensity-modulated radiation therapy, SBRT: stereotactic body radiation therapy, SVCS: superior vena cava syndrome

Not specific: patients did not have specific symptoms that warranted immediate palliative radiation

*: Ablative thoracic radiation was planed for this patient and ended up as palliative radiation due to the development of grade 3 radiation pneumonitis

^#^: Thoracic radiation was considered ablative with a biologic effective dose (BED)≥60 Gy_10_ for IMRT and with a BED ≥100 Gy_10_ for SBRT, adapted from previous studies[[1](#_ENREF_1),[2](#_ENREF_2)]

**Reference**:

[1] Ashworth AB, Senan S, Palma DA, et al. An individual patient data metaanalysis of outcomes and prognostic factors after treatment of oligometastatic non-small-cell lung cancer. Clinical lung cancer 2014;15:346-355.

[2] Gomez DR, Blumenschein GR, Jr., Lee JJ, et al. Local consolidative therapy versus maintenance therapy or observation for patients with oligometastatic non-small-cell lung cancer without progression after first-line systemic therapy: a multicentre, randomised, controlled, phase 2 study. The Lancet. Oncology 2016;17:1672-1682.
